# Potato- An Important Source of Nutritional Kynurenic Acid

**DOI:** 10.1007/s11130-012-0283-3

**Published:** 2012-03-06

**Authors:** Michal P. Turski, Piotr Kamiński, Wojciech Zgrajka, Monika Turska, Waldemar A. Turski

**Affiliations:** 1 Department of Toxicology, Institute of Agricultural Medicine, Jaczewskiego 2, 20-950 Lublin, Poland; 2 National Research Institute, Potato Breeding Zamarte, Plant Breeding and Acclimatization Institute, Zamarte 33, 89-430 Kamień Krajeński, Poland; 3 Department of Experimental and Clinical Pharmacology, Medical University, Jaczewskiego 8, 20-090 Lublin, Poland

**Keywords:** Crisps, French fries, Kynurenic acid, Potato

## Abstract

Kynurenic acid (KYNA) is a metabolite of tryptophan which is formed along the kynurenine pathway. KYNA may possess neuroprotective, anti-inflammatory, antioxidant and antiproliferative properties. This study measured the concentration of KYNA in various varieties of potatoes and products made from potatoes. KYNA content was determined by means of the high-performance liquid chromatography with fluorescence detection. KYNA was found in all 16 studied varieties of potato tubers in amounts varying from 0.239 to 3.240 μg/g dry weight. The content of KYNA in potato tubers declined during long-term storage. The content of KYNA in French fries varied from 0.100 to 0.646 μg/g dry weight. KYNA content in potato crisps was 0.478 and 0.576 μg/g dry weight. Hence, all in all, we concluded that the amount of KYNA potentially delivered to the human body in potatoes and various foods produced from potatoes is high and might be compared to the amount of KYNA present in a maximum daily dose of popular herbs and herbal medicines.

## Introduction

Kynurenic acid (KYNA) is an oxidative pathway metabolite of tryptophan which is formed along the kynurenine pathway. Its presence was first demonstrated in urine by Liebig in 1853 [1]. KYNA is an endogenous antagonist of ionotropic glutamate receptors and α7 nicotinic acetylcholine receptor which exerts an anticonvulsant and neuroprotective activity [2–5]. Furthermore, KYNA exerts a modulatory effect on the neurotransmission in the brain, based on electrophysiological studies [3, 6]. The possible role of KYNA in the central nervous system has been recently reviewed by Wonodi & Schwarcz [7], Vamos et al. [8] and Erhardt et al. [9].

Nevertheless, identification of KYNA and its potential role outside the brain has aroused interest in recent years. KYNA was found in urine, serum, amniotic and synovial fluid, where its concentration varied from 0.02 to 0.8 *μM* [10–13]. Interestingly, KYNA was also found in rat intestinal fluid. It was stated that its content increases along the small intestine, reaching a concentration of 16 *μM* in its distal part [14]. It has been shown recently that it is also an agonist of the G-protein-coupled receptor GPR35. Interestingly, GPR35 receptor is present in a significant quantity in the gastrointestinal tract [15]. Furthermore, KYNA in a concentration found in the digestive system may interact not only with GPR35 receptor, but also with N-methyl-D-aspartate (NMDA) receptors and α7 nicotinic receptors [14]. The role of KYNA in the gastrointestinal tract is relatively unknown as the studies concerning it are at the initial stage. Nevertheless, it has already been shown that KYNA possesses anti-ulcerative properties [16, 17]. Furthermore, it is known that KYNA shows anti-inflammatory properties and antagonizes hypermotility of the intestine in an experimentally induced colon obstruction [18]. KYNA, as an antagonist of NMDA receptors, decreases motility and inflammatory activation in the early phase of acute experimental colitis in rats [19]. Moreover, it has been stated that KYNA decreases proliferation of colon derived cancer cells *in vitro*, and that its content in the intestinal mucus is increased in patients with diagnosed colon lesions such as adenoma or adenocarcinoma [20]. Finally, it has been shown recently that KYNA possesses antioxidative properties [21].

In general, these findings suggest a potential role of KYNA in the regulation of the gastrointestinal tract. It is worth mentioning that one of the sources of KYNA in the intestine might be natural food and nutritional products derived from it [14, 22]. In our previous study it was proved that KYNA is present in food and honeybee products. KYNA was found in all tested samples. The highest concentration of KYNA was obtained from samples of various honeybee products, such as propolis, multiflorous honey and bee pollen. Nevertheless, a high concentration of KYNA was also found in fresh broccoli and potato. Interestingly, potato contains 10 times more KYNA than meat, egg, carrot, tomato or paprika [23]. It should be noted that potato is one of the world’s most important food crops, together with maize, wheat, rice and soy bean. The world’s potato production exceeded 325 million tonnes in 2007, and consumption was more than 31 kg *per capita* in 2005, according to FAO’s data.

16 varieties of potatoes were chosen in order to conduct the study. The choice of varieties was consulted with specialists from the Plant Breeding and Acclimatization Institute in Zamarte. All varieties were edible and grown in the same conditions such as the same climate and soil. They differed in terms of starch content, maturity and storage ability.

Potential health effects and promising medicinal uses of potato-derived compounds and products were recently reported [24–27]. It is known that the concentration of some nutrients in potatoes decreases during a long-term storage [29–32]. Therefore, it was investigated whether storage time influences the concentration of KYNA. Finally, the content of KYNA in French fries, crisps and flour was also examined to see whether they can be a valuable source of KYNA since in some cases the consumption of potato-related foods might even be higher than the consumption of potatoes.

Summing up, all the reasons stated above made us examine concentration of KYNA in various specimen of potatoes and foods which are produced from them.

## Materials and Methods

### Standard and Reagents

Kynurenic acid (KYNA) standard (98.5% purity, determined by HPLC) used for the quantitative analysis of KYNA was purchased from Sigma (St. Louis, MO, USA). All high performance liquid chromatography (HPLC) reagents were purchased from J.T. Baker (Deventer, Netherlands) and were of the highest available purity: zinc acetate (analytical purity 100%), sodium acetate (analytical purity 100%, assay by non-aqueous titration), water (HPLC gradient grade), acetonitrile (HPLC ultra gradient grade, purity determined by GC) and isopropyl alcohol (99.5%). Hydrochloric acid (J.T. Baker, Deventer, Netherlands), potassium hydroxide and trichloroacetic acid (both POCH Gliwice, Poland) were of an analytical grade. For purification of the extracts obtained from the plant material and food samples and for extraction of KYNA, cation exchange resin Dowex 50 WX4-400 purchased from Sigma (St. Louis, MO, USA) was used.

### Materials

All 16 studied potato (*Solanum tuberosum*) varieties were grown and harvested in the Plant Breeding and Acclimatization Institute in Zamarte, Poland. All varieties were edible. They were cultivated in accordance with planting guidelines under comparable soil and weather conditions. The varieties differed in terms of starch content, maturity and storage ability (see Table 1 for details). All investigated potatoes were harvested in September 2009. They were stored under optimal, standard conditions. Potatoes were kept in an appropriate storage facility with good mechanical ventilation. Temperature and humidity were controlled. Storage of potatoes was performed in the following phases: drying of surface moisture (one week temperature 12–18 °C, humidity 75–95%); wound healing phase (one week temperature 12–18 °C, humidity 90–95%); a staged cooling phase (four weeks temperature reduction 0.2–1.0 °C/day, humidity 90–95%); holding phase (temperature 4–6 °C, humidity 90–95%); and reconditioning phase, during which the tubers were slowly warmed (temperature 10–15 °C, humidity 75–80%).

**Table 1:** Content of kynurenic acid in potatoes

Potato varieties	Kynurenic acid content [μg/g dry weight]	Kynurenic acid content [μg/g wet weight]	Changes in kynurenic acid content during storage			Potato characteristics ^a^		
			October 2009 [%]	January 2010 [%]	April 2010 [%]	Starch content [%]	Maturity	Storage ability
Finezja	3.240 ± 0.505	0.648 ± 0.101	100	91	73*	14.5	medium early	medium
Justa	2.546 ± 0.156	0.491 ± 0.030	100	109	70*	13.5	very early	good
Milek	1.349 ± 0.361	0.239 ± 0.064	100	81	44*	12.4	very early	very good
Etola	0.939 ± 0.387	0.153 ± 0.063	100	96	73*	16.6	early	medium
Elanda	0.912 ± 0.060	0.168 ± 0.011	100	81*	17*	12.9	medium early	medium
Denar	0.832 ± 0.299	0.139 ± 0.050	100	77*	22*	11.7	very early	very good
Oman	0.690 ± 0.109	0.145 ± 0.023	100	46*	29*	14.7	early	good
Lord	0.635 ± 0.059	0.107 ± 0.010	100	94	39*	11.7	very early	very good
Tetyda	0.622 ± 0.203	0.104 ± 0.034	100	40*	8*	11.7	medium early	good
Medea	0.581 ± 0.209	0.122 ± 0.044	100	26*	25*	14.7	late	poor
Flaming	0.517 ± 0.203	0.107 ± 0.042	100	64*	36*	14.5	very early	medium
Eugenia	0.484 ± 0.093	0.083 ± 0.016	100	80	63*	12.0	early	medium
Korona	0.479 ± 0.186	0.080 ± 0.031	100	29*	15*	11.4	early	poor
Aruba	0.436 ± 0.122	0.089 ± 0.025	100	7*	4*	14.3	early	very good
Benek	0.358 ± 0.066	0.071 ± 0.013	100	74*	49*	13.9	medium early	medium
Zagloba	0.239 ± 0.066	0.040 ± 0.011	100	54*	40*	11.7	late	very good

Kynurenic acid content was determined in October 2009, January 2010 and April 2010. Data are presented as a mean ± a standard error of the mean (SEM). Changes in kynurenic acid content are presented as a percentage of substance content measured in October. Statistical analysis was performed using one-way ANOVA with Tukey *post-hoc* test

*P < 0.05 was considered statistically significant versus content of kynurenic acid in October 2009

^a^Potato characteristics according to the data from Plant Breeding and Acclimatization Institute, National Research Institute, Potato Breeding Zamarte, Poland

Sweet potatoes (*Ipomoea batatas*), potato flour (starch), wheat flour, French fries and crisps were of commercial origin. The following French fries were used: Plain123 French fries (McCain, Chociwel, Poland); Zigzag extra crispy and crinkle oven French fries (Aviko B.V., Steenderen, Holland); Oven Crinkles French fries (Farm Frites Poland SA, Lębork, Poland).

The following crisps were used: Cheetos cheese, Lays natural salted, Star prazynki (all from Frito Lay Poland, Grodzisk Mazowiecki, Poland) and Crunchips x-cut salted, Peppies Snack Bacon (both from the Lorenz Bahlsen Snack-World, Sady/Pozna•, Poland).

### Preparation of Samples for KYNA Determination

Potato tubers were cleaned of remaining soil, weighed and ground up. Distilled water (1:4, w/v) was added and they were then homogenized. Samples containing 1 ml of homogenate were collected. KYNA was added to some of the samples as an internal standard. All samples, with or without added KYNA solution, were acidified with 100 μl of 2.4 *N* trichloroacetic acid. Denaturated proteins were removed by centrifugation (4,000 rpm, 5 min). Supernatant was collected for further experiments.

Samples of flour were weighed and distilled water was added to them (1:9 w/v). They were then homogenized. Samples containing 2 ml of homogenate were collected. KYNA was added to some of the samples as an internal standard. Then, 100 μl of 2.4 *N* KOH was added to all samples, with or without added KYNA solution. Samples were then vortexed and acidified with 200 μl of 2.4 *N* trichloroacetic acid. Denaturated proteins were removed by centrifugation (4,000 rpm, 5 min). Supernatant was collected for further experiments.

French fries were weighed and both distilled water (1:5, w/v) and Tween 80 (Sigma; 1 drop/5 ml) were added afterwards. They were then homogenized. Samples containing 2 ml of homogenate were collected. KYNA was added to some of the samples as an internal standard. Then, 100 μl of 2.4 *N* KOH was added to all samples, with or without added KYNA solution. Samples were then vortexed and acidified with 200 μl of 2.4 *N* trichloroacetic acid. Denaturated proteins were removed by centrifugation (4,000 rpm, 5 min). Supernatant was collected for further experiments.

Crisps were weighed and both distilled water (1:10, w/v) and Tween 80 (Sigma; 1 drop/5 ml) were added afterwards. Samples were then vortexed and heated to 40 °C. Afterwards, they were homogenized. Samples containing 1 ml of homogenate were collected. KYNA was added to some of the samples as an internal standard. Then, 30 μl of 2.4 *N* KOH was added to all samples, with or without added KYNA solution. Samples were vortexed and acidified with 100 μl of 2.4 *N* trichloroacetic acid. Denaturated proteins were removed by centrifugation (4,000 rpm, 5 min). Supernatant was collected for further experiments.

### Determination of KYNA

KYNA was isolated and determined according to the methods described previously [5, 28]. Samples (1 ml) were acidified with 0.1 *N* HCl and applied to the columns containing cation exchange resin Dowex 50 prewashed with 0.1 *N* HCl. Subsequently, the columns were washed with 1 ml *(an elsewhere)* of 0.1 *N* HCl and 1 mL of water. The fraction containing KYNA was eluted with 4 mL of water. The eluate was subjected to HPLC and KYNA was detected fluorometrically (Hewlett Packard 1050 HPLC system: ESA catecholamine HR-80, 3 μm, C18 reverse-phase column, mobile phase: 250 *mM* zinc acetate, 25 *mM* sodium acetate, 5% acetonitrile, pH 6.2, flow rate of 1.0 ml/min; fluorescence detector: excitation 244 nm, emission 398 nm). Mobile phase was filtered through a 0.2 μm membrane filter (Alltech Associates, USA) and degassed by ultrasonic equipment for 30 min. The injection volume was 100 μl. Separation was achieved at an ambient temperature. The Chemstation software was employed to control HPLC system and process chromatographic data.

### Statistical Analysis

Data were presented as a mean value and a standard error of the mean (SEM). Statistical analysis was performed using one-way ANOVA with Tukey *post-hoc* test. *P < 0.05 was considered statistically significant.

## Results

KYNA was found in all 16 studied varieties of potato tubers. Its content varied from 0.239 to 3.240 μg/g dry weight. The highest content of KYNA was found in the Finezja, Justa and Milek varieties. The lowest amount of KYNA was recorded in Aruba, Benek and Zagloba varieties.

KYNA content in potato tubers declined during long-term storage. The content of KYNA in Tetyda and Aruba varieties declined by 92 and 96% in comparison to the initial content recorded after harvest, respectively. The lowest decline, by 27%, was recorded in both Finezja and Etola varieties. No correlation between starch content, maturity, storage ability and the concentration of KYNA in potatoes was found. Detailed data expressed on both wet and dry weight are presented in Table 1.

The content of KYNA measured in sweet potato was 0.024 μg/g dry weight. KYNA content in French fries varied from 0.100 to 0.646 μg/g dry weight (Table 2). The content of KYNA in crisps produced from potatoes–Crunchips and Lay’s was 0.576 and 0.478 μg/g dry weight, respectively. In crisps containing starch and potato flakes–Star chips–the amount of KYNA was 0.063 μg/g dry weight. KYNA content was 0.032 μg/g dry weight in crisps containing starch and wheat flour. In crisps produced from corn, KYNA content was 0.165 μg/g dry weight. KYNA was found in both potato flour and wheat flour in amount of 0.035 and 0.008 μg/g dry weight, respectively. Detailed data expressed on both wet and dry weight are presented in Table 2.

**Table 2:** Kynurenic acid content and estimated intake of kynurenic acid in potatoes and selected nutritional products

Food	Product	Kynurenic acid content [μg/g dry weight]	Kynurenic acid content [μg/g wet weight]	Average consumption^i^ [g wet weight]	Kynurenic acid intake [μg]
Fresh potatoes	Potato–Finezja	3.240 ± 0.505	0.648 ± 0.101	100	64.8
	Potato–Medea	0.581 ± 0.209	0.122 ± 0.044	100	12.2
	Potato–Zagloba	0.239 ± 0.066	0.040 ± 0.011	100	4.0
	Sweet potato	0.024 ± 0.0005	0.005 ± 0.001	100	0.5
French fries	Zigzag^a^	0.646 ± 0.012	0.160 ± 0.003	100	16.0
	Plain123^b^	0.364 ± 0.006	0.121 ± 0.002	100	12.1
	Oven Crinkles^c^	0.100 ± 0.003	0.035 ± 0.001	100	3.5
Crisps	Cheetos^d^	0.165 ± 0.001	0.157 ± 0.001	30	4.7
	Lay’s^e^	0.478 ± 0.003	0.463 ± 0.003	30	13.9
	Star^f^	0.063 ± 0.002	0.060 ± 0.002	30	1.8
	Crunchips^g^	0.576 ± 0.016	0.552 ± 0.015	30	16.5
	Peppies Snack Bacon^h^	0.032 ± 0.001	0.031 ± 0.001	30	0.9
Flour	Potato flour (starch)	0.035 ± 0.003	0.032 ± 0.003	100	3.2
	Wheat flour	0.008 ± 0.001	0.007 ± 0.001	100	0.7

Data are presented as a mean ± a standard error of the mean (SEM)

Main ingredients

^a^potatoes, vegetable oil, modified starch, rice flour

^b^potatoes, vegetable oil

^c^potatoes, vegetable oil, dextrose

^d^corn, vegetable oil

^e^potatoes, vegetable oil, salt

^f^potato starch, vegetable oil, potato flakes, salt

^g^potatoes, vegetable oil, salt

^h^wheat flour, potato starch, vegetable oil, salt

^i^calculated basing on subjective assumptions of a daily consumption of potatoes, French fries or flour–100 g (one portion of potatoes or French fries served in restaurants and assumed average daily consumption of flour) and crisps–30 g (one small pack of crisps)

Intake of KYNA in potatoes and studied potato-related foods was calculated due to an estimated daily consumption of these products: potatoes, French fries or flour–100 g (one portion of potatoes or French fries served in restaurants and assumed average daily consumption of flour) and crisps–30 g (one small pack of crisps). The highest daily intake was estimated in the case of consumption of Finezja potatoes–64.8 μg/100 g wet weight, Crunchips potato crisps–16.5 μg/30 g wet weight and Zigzag French fries–16.0 μg/100 g wet weight. The lowest daily intake was calculated for the consumption of sweet potatoes–0.5 μg/100 g wet weight, food containing wheat flour–0.7 μg/100 g wet weight and Peppies crisps–0.9 μg/30 g wet weight. Detailed data expressed on both wet and dry weight are presented in Table 2.

## Discussion

Recently, we found that KYNA is present in potatoes in a relatively significant amount [23]. In the present study we confirmed that KYNA is present in potato tubers by examining 16 varieties of potatoes. All tested potatoes were cultivated in the Plant Breeding and Acclimatization Institute in Zamarte which specializes in the breeding and production of seed material. The content of KYNA in the examined potato tubers varied from 0.239 to 3.240 μg/g dry weight. The highest amount of KYNA was found in the Finezja and Justa varieties. The lowest amount of KYNA was found in Benek and Zagloba varieties. The reasons for differences in the concentration of KYNA between the varieties of potatoes investigated are unknown. All the varieties were edible and grown in the same conditions such as climate and soil. They differed in terms of starch content, maturity and storage ability but there was no correlation between these parameters and the concentration of KYNA in potatoes. Therefore, there is a need for further research concerning the reasons for differences in KYNA content among varieties of potatoes. Furthermore, we also investigated the influence of storage of potatoes on the content of KYNA in potato tubers. Potatoes were stored in optimal conditions, with regard to experts’ guidelines. Our experiments proved that the concentration of KYNA decreases as the storage period is lengthened. In some of the investigated potato varieties, such as Finezja and Etola, the amount of KYNA decreased slightly, by about 30%. Nevertheless, we also discovered that some potato varieties, such as Tetyda and Aruba, lost more than 90% of their initial KYNA content. It is worth mentioning that changes in the content of various nutrients in potatoes are a commonly known phenomenon. For example, a decrease in concentration of reducing sugar [29], starch [30] and vitamin C [31, 32] in potatoes during storage has been described.

The role of KYNA in potato tubers is not known. Furthermore, a metabolite created from KYNA has also not been discovered yet. It has been suggested that KYNA might be transformed into quinaldic acid [33]. Nevertheless, it has not been proved yet whether this process occurs in potato tubers.

KYNA is thought to possess neuroprotective properties in the brain. However, it is vital to point out that the blood–brain barrier reduces its application as a drug acting on the central nervous system. Numerous scientific papers describing valuable properties of KYNA outside the brain have been published recently. Its antiulcer [16, 17] and anti-inflammatory properties, as well as ability to regulate intestinal motility, have been proved [18, 19]. Furthermore, the beneficial role of KYNA expressed in colitis ulcerosa [19] has been suggested. Finally, there is very recent information that KYNA might possess antioxidative [21] and antiproliferative properties [20].

Bearing in mind all the properties of KYNA mentioned above, it seems vital to take the intake of KYNA from food into consideration. Potatoes contain one of the highest concentrations of KYNA among food. It is worth mentioning that only various kinds of honey contain more KYNA than potatoes [23, 34, 35]. Since potatoes are widely known as a constituent of an everyday diet, not only in the form of tubers but also other types of food, we measured the concentration of KYNA in some potato-related products. As expected, KYNA was found in French fries. Its content varied from 0.100 to 0.646 μg/g dry weight. Unfortunately, the varieties of potato used for the production of the fries were not specified by the producer.

A very popular food produced from potatoes are crisps. They are very popular not only among adolescents, but also among adults. It is, however, worth mentioning that not all kinds of crisps are produced from potatoes. This is the reason why there are such significant differences among various crisps. We have proved that the concentration of KYNA in crisps produced from potatoes is high. Yet, other kinds of crisps, such as those produced from corn contain a few times less KYNA in comparison to potato crisps. Other snacks, such as puffs produced from potato flakes and starch, contain a significantly lower amount of KYNA than potato crisps. Moreover, crisps produced from wheat flour and starch do not contain as much KYNA as potato crisps.

We have investigated the content of KYNA in potato flour and compared the achieved results to the content of KYNA in potato tubers. Indeed, the concentration of KYNA in potato flour is lower than the concentration of KYNA in potato tubers. Since potato flour is commonly used to produce food it might be advisable to consider a change in the production method of potato flour from potatoes, in order to make the content of KYNA in the end-product higher. Moreover, we found that KYNA content in wheat flour is about five times lower than in potato flour.

Our results indicate that potato tubers and other types of food produced from potatoes might be an important source of KYNA in everyday diet. Based on our estimations, when taking a daily intake of potatoes and food produced from potatoes into account, the highest concentration of KYNA consumed by our organisms comes from potato tubers, mainly due to the high content of KYNA in them as well as a high amount of digested potatoes. Crisps produced from potatoes occupy the second place and are the best source of KYNA among potato-related products in countries where potato tubers are not the basic component of an everyday diet. The evaluation of the amount of KYNA in other types of food, such as puffs produced from potatoes, crisps produced from wheat flour or crisps produced from corn, demonstrated that the amount of KYNA delivered to human organisms with those kinds of food is much lower. The amount of KYNA delivered to the organism in potatoes and food produced from potatoes might be compared to the amount of KYNA in a maximum daily dose of popular herbs and herbal medicines. We have recently shown that there is a high concentration of KYNA in St. John’s Wort, which is commonly used for depression [36] and many other conditions. The amount of KYNA in a maximum daily dose of St. John’s Wort is 30.38–33.60 μg [37]. Our current results indicate that a similar concentration of KYNA can be found in one standard serving of potato tubers (100 g wet weight) or one pack of crisps (30 g wet weight). It appears that the content of KYNA in other similar types of food harvested, produced and consumed in other parts of the world, such as rice [23] or sweet potatoes, is significantly lower.

Therefore, potatoes seem to be a rich source of KYNA for humans. Therefore, it seems reasonable to increase the supply of KYNA to the organism due to the proper choice of various varieties of potatoes, which contain high amounts of KYNA and do not lose a lot of KYNA during the storage period.

## Abbreviations

KYNA: Kynurenic acid
NMDA: N-methyl-D-aspartic acid
